# Examination of Driver Visual and Cognitive Responses to Billboard Elicited Passive Distraction Using Eye-Fixation Related Potential

**DOI:** 10.3390/s21041471

**Published:** 2021-02-20

**Authors:** Yongxiang Wang, William Clifford, Charles Markham, Catherine Deegan

**Affiliations:** 1 School of Electrical and Electronic Engineering, City Campus, Technological University Dublin, D08 NF82 Dublin 8, Ireland; catherine.deegan@tudublin.ie; 2 Department of Computer Science, National University of Ireland, Maynooth, W23 F2H6 Kildare, Ireland; william.clifford@mu.ie (W.C.); charles.markham@mu.ie (C.M.)

**Keywords:** driver distraction, billboard distraction, driving simulator, eye-fixation related potential (EFRP), EEG, event-related potentials (ERPs), eye tracking, human information processing

## Abstract

Distractions external to a vehicle contribute to visual attention diversion that may cause traffic accidents. As a low-cost and efficient advertising solution, billboards are widely installed on side of the road, especially the motorway. However, the effect of billboards on driver distraction, eye gaze, and cognition has not been fully investigated. This study utilises a customised driving simulator and synchronised electroencephalography (EEG) and eye tracking system to investigate the cognitive processes relating to the processing of driver visual information. A distinction is made between eye gaze fixations relating to stimuli that assist driving and others that may be a source of distraction. The study compares the driver’s cognitive responses to fixations on billboards with fixations on the vehicle dashboard. The measured eye-fixation related potential (EFRP) shows that the P1 components are similar; however, the subsequent N1 and P2 components differ. In addition, an EEG motor response is observed when the driver makes an adjustment of driving speed when prompted by speed limit signs. The experimental results demonstrate that the proposed measurement system is a valid tool in assessing driver cognition and suggests the cognitive level of engagement to the billboard is likely to be a precursor to driver distraction. The experimental results are compared with the human information processing model found in the literature.

## 1. Introduction

Billboards are a popular means of advertising. Traditionally, they are large, printed signs placed in the drivers view at the roadside. Recent advances have introduced digital billboards which are large electronic image displays that present single or multiple, static, or dynamic advertisements to convey information. Globally, billboards have been developing rapidly on road networks. Locations with high traffic volume are most attractive to advertisers [1]. As a result, driver attention is being demanded by these billboards, which may affect driver attention given to the roadway. Standards and regulations have been published in both Europe and the US to standardise the design and production of billboards. Billboard related road collisions have been shown to be a cause of traffic collisions [2,3]. The research studies that investigated billboards related driver distraction has shown that billboards can increase crash risk [4]. This study proposed to use a combined EEG and eye tracking system and a customised driving simulator to investigate driver distraction caused by billboards. By comparing drivers’ visual and cognitive responses to eye-fixation behaviour on billboards and eye-fixation that assists driving, we found differences in their brain cognitive processes.

Billboards can be broadly divided into two subcategories: active and passive. The active billboards change their content regularly, one or more advertisements can be seen by the driver over a short period of time. The passive billboard contains a single advertisement. The active billboard can include digital billboards or signs that change displays mechanically (e.g., ‘rollerbar’ or ’tri-vision’ billboards). Digital billboards can be potentially more distracting due to their size, colour and prominent locations. Large outdoor digital billboards usually appear alongside roadways. It might therefore be expected that they would contest for attention from the driving task. The National Highway Traffic Safety Administration (NHTSA) has conducted a research study based on data collected using eye tracking tools, their results show digital billboards affect visual attention of drivers, which potentially leads to road accidents [5].

Determining the effect of billboards on driver distraction presents a challenging problem. Two early reports investigated and reviewed the relationship between road crash rate and external distractions (including billboards) from various accident investigations and accident causation databases [3,6]. Both studies elaborated that many previous studies have not been able to show a link between crash rate and the presence of billboards. More recent studies have also not found a significant difference in crash prevalence due to roadside advertising [7,8,9]. Furthermore, research studies revealed that drivers may be unlikely to identify billboard distraction as a cause of a crash as they feel it might expose their liability [3,6]. Additionally, real accident data does not provide information about the cognitive process in the driver prior to the accident. However, laboratory experiments provide a controlled environment to ascertain how billboards might impair driving performance. Johnston and Cole [10] found that significant decrements in task performance are produced under conditions of critical load by the presentation of non-driving-related signs. Luoma [11] developed and tested a simulation method that found billboards distract and reduce the conscious perception of the traffic signs. Clark and Davies [12] found that non-driving-related signs delay the responses to road signs in a simulated driving task. Bendak and Al-Saleh [13] found that driving performance (lane drifting and recklessly crossing dangerous intersections) was worse on a road with advertising signs compared with no advertising signs. A recent study found both content of advertising and billboard location to drivers’ viewing field have high influence on driver distraction [14].

In most driver distraction studies, eye trackers have been used to track the eye movement in order to investigate driver visual behaviour. Eye movement measurements and glance duration measurements are commonly used techniques. other studies divided visual behaviour into four categories, (1) gaze variability (GV), glance pattern activity (GPA), and percentage of time spent glancing at the forward roadway; (2) glances at unexpected drive-relevant stimuli; (3) glances at expected drive-relevant stimuli; and (4) glances at billboards [15]. Edquist et al. [16] found that roads with advertising billboards delay the driver response time to road signs and lead to less time fixating on the road ahead compared to roads without billboards. Beijer et al. [17] found that significantly longer glances were present for active signs when compared to passive signs. Crundall et al. [18] reported the height level of roadside advertisements as a factor increasing the number of driver’s eye fixations. Lee et al. [19] measured the eyeglances behaviour found digital billboards attract more driver’s attention, which caused a significantly greater impairment to driving performance when compared with static billboards. Megías et al. [20] found that emotional advertisements elicited a larger number of eye fixations compared to neutral advertisements. Chattington et al. [21] found that full motion video billboards were associated with more glances away from the road than static billboards. Smiley et al. [22], Hudák and Madleñák [23,24] demonstrated driver’s gaze and fixation durations on billboards for relatively long periods. Moreover, in a real-world driving study, Dukic et al. [25] found drivers spend a greater number of fixations and longer fixation durations when driving past electronic billboards compared to other road signs.

The use of an eye tracker can also measure pupil diameter and dilation change. They can reflect eye workload in a driving task. Schwalm et al. [26] used pupillometry as an index of real-time physiological indicator of cognitive workload. The study examined the changes in the size of the human pupil showing a correlation with eye workload and supported the use of pupillometry as an efficient tool for distraction study. Several other studies have used the rate of pupil area change while studying workload of drivers in driving tasks [27,28,29].

Researchers also give some attention to the driver’s age and experience related to billboard distraction. Edquist et al. [16] and Stavrinos et al. [30] found both inexperienced and older drivers may be more vulnerable to billboard distractions. However, Topolsek et al. [31] found that age was not associated with the number of times billboards are detected by the driver.

In addition to using an eye tracker, electroencephalography (EEG) and, more recently, event-related potential (ERP) have been used in the detection of driver distraction. EEG is a non-invasive device that uses a computer to record the small electrical activity of the brain through electrodes placed on the scalp. The EEG signal has the ability to probe driver behaviour in response to workload [32,33] and driver fatigue [34,35]. ERP technique measures brain response that is the direct result of a specific sensory, cognitive or motor event. It is comprised of several temporally separated underlying components. Every component is associated with several brain functions or the timing of information processing [36]. In driver distraction research, the use of ERP is usually used to measure the amplitude and latency of one or several components of the EEG signal. For example, a study uses of negative slow wave (NSW), the most negative-going ERP in the range 430–995 ms at electrodes Fz and Cz, to assess the allocation of neural resources under single and dual-task conditions [37]. The study found a reduced NSW amplitude in dual-task compared to single-task conditions and suggested driver shifted cognitive resources from the primary driving task to processing the distracting stimuli. In another study comparing P300 amplitudes in relation to difficulty in the act of driving, it was found that increases in difficulty were related to decreases in the P300 amplitude [38].

Eye-fixation related potential (EFRP) is another type of ERPs, in which the triggering event is based on eye fixation. Machii et al. [39] and Terada et al. [40] found the amplitude of the lambda response of EFRP decreases as the secondary task becomes more difficult. Both studies reveal that decreased visual attention was caused by distractions to the secondary task. Ahlström et al. [41] discovered night driving decreases cortical responsiveness to visual stimuli using EFRP. Renold et al. [42] found differences in early evoked activity between target and non-target items in a driving simulator visual research study.

Very few studies have investigated billboard related driver distraction using EEG/ERP. Hudák and Madleñák [23] reported an increased instantaneous excitement using EEG when driver attention is competed for by billboards, high or unclear traffic on the roads or police evidence. Haak et al. [43] use EEG to detect high-frequency eye blinks triggered by attention-seeking billboards. Yang et al. [44] measured EEG alpha power in response to increased driver mental workload. This was done by comparing the response to a single-board sign and multi-board sign.

The reviewed literature suggests that it is feasible to use EEG/ERP to assess driver distraction. In this present study, we use a synchronised EEG and eye-tracking system and a customised driving simulator to measure driver visual and cognitive response to billboards, which passively distracted driver attention. Additionally, the literature has demonstrated the capability of using EFRP in driver distraction studies. We utilise this technique to investigate driver early brain response for early visual processing and subsequent scene processing after visual information is registered. The scientific contribution of this study is that we have proposed and developed an experimental apparatus that uses combined EEG and eye tracking, as well as corresponding methods that can detect driver distraction caused by the billboard. This research provides a unique approach of synchronising EEG and eye tracking to monitor driver distraction in a complex dynamically changing scene.

## 2. Methods

In this section, we provide a detailed description of the experimental method using synchronised EEG and eye tracking and customised driving simulator. The structure of a customised driving simulator, the experimental procedure, the profile of the participants, the hardware used for data recording are outlined. The choice of eye-fixation events and the analysed ERP components are discussed.

### 2.1. Driving Simulator

**Figure 1:**
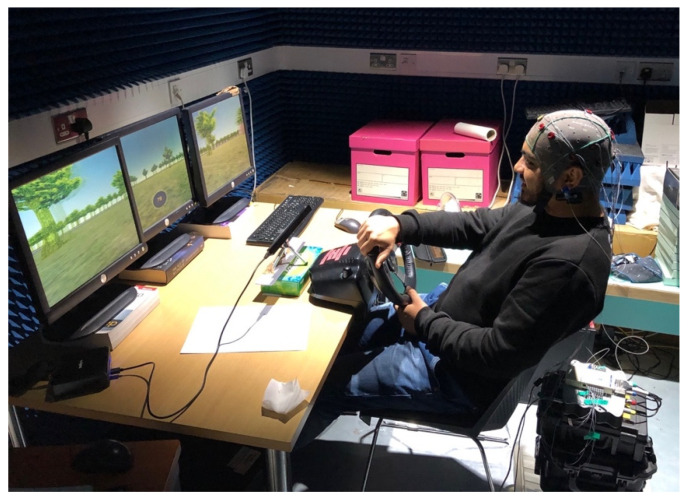
A participant undertaking a test drive with the driving simulator.

The experiment setup was built around a driving simulator developed using the Unity™ 3D game engine [45]. The Unity simulator consists of four basic components, a two-lane highway road asset, a car asset, road sign assets and billboard assets. The car asset model, BMW M3, was selected as it provided a realistic model and included a physics engine that modelled the cars behaviour [46]. The Unity simulator runs on a Windows 10 based computer, containing an Intel™ Quad-Core Xeon processor and a Nvidia™ graphic card, displays on a triple screen (three 1024 × 768 monitors connect to a Matrox™ TripleHead2Go converter interface to a computer act as a single 3072 × 768 monitor). In order to control the car in the driving environment, a Logitech™ G27 steering wheel with force feedback and primary pedals were provided. The driver sits facing the centre screen. The simulator was configured so that there was no other traffic on the road. Figure 1 shows a participant undertaking a test drive with the driving simulator.

**Figure 2:**
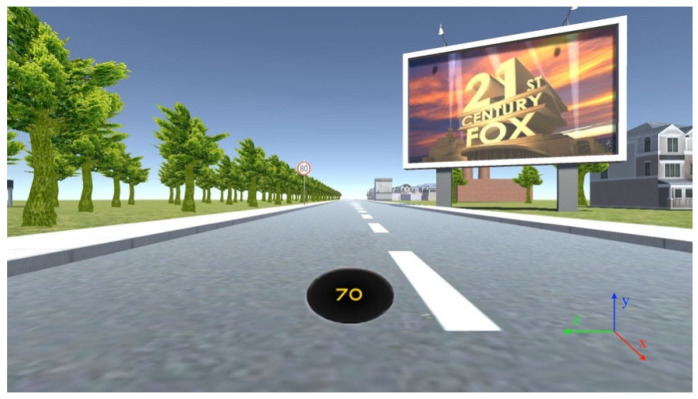
A screenshot showing the first-person perspective driving in the driving simulator. The right bottom corner 3D axis legend is added separately.

The driving simulator used a first-person view, so as to allow a full view of the road with car hood and window out of view (Figure 2). The car in the driving simulator was configured with a set of pre-defined waypoints to allow the car to drive along a route defined by customised lane position on the road. The steering wheel was disabled but free to move in the experiment. This was done to remove unnecessary distractions caused by the steering control. The accelerator pedal was used by the driver to control the driving speed. The dashboard of the car included a digital speedometer. The maximum driving speed is limited at 90 km/h.

**Figure 3:**
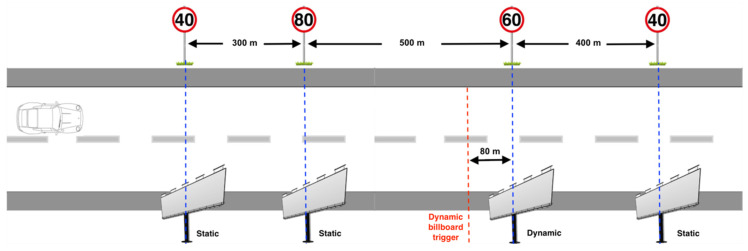
An illustration of the experiment design.

There were a total number of 50 test regions each containing a speed sign and digital billboard. The speed signs were located on the left side of the road and the billboard on the right. Each speed sign and digital billboard pair was positioned on the same *x*-axis as shown in Figure 2 and Figure 3. The distance from the current pair of speed sign and digital billboard to the next pair was defined according to the speed limit displays on the speed sign (see Figure 2). For instance, if the current speed sign is limited at 40 km/h, then the distance from the current pair of speed sign and digital billboard to the next pair is at 300 m. For the speed limit at 60 km/h, the distance was set at 400 m. For the speed limit at 80 km/h, the distance was set at 500 m. There is no particular reason to choose these values, but 300-unit distance is guaranteed the next digital billboard is not recognisable when the driver is located at the current digital billboard. Another consideration was the length of the driving time should not be too long, to avoid driver fatigue. The speed limit values were set randomly but chosen to avoid signs having the same speed consecutively (see Table 1).

**Figure 4:**
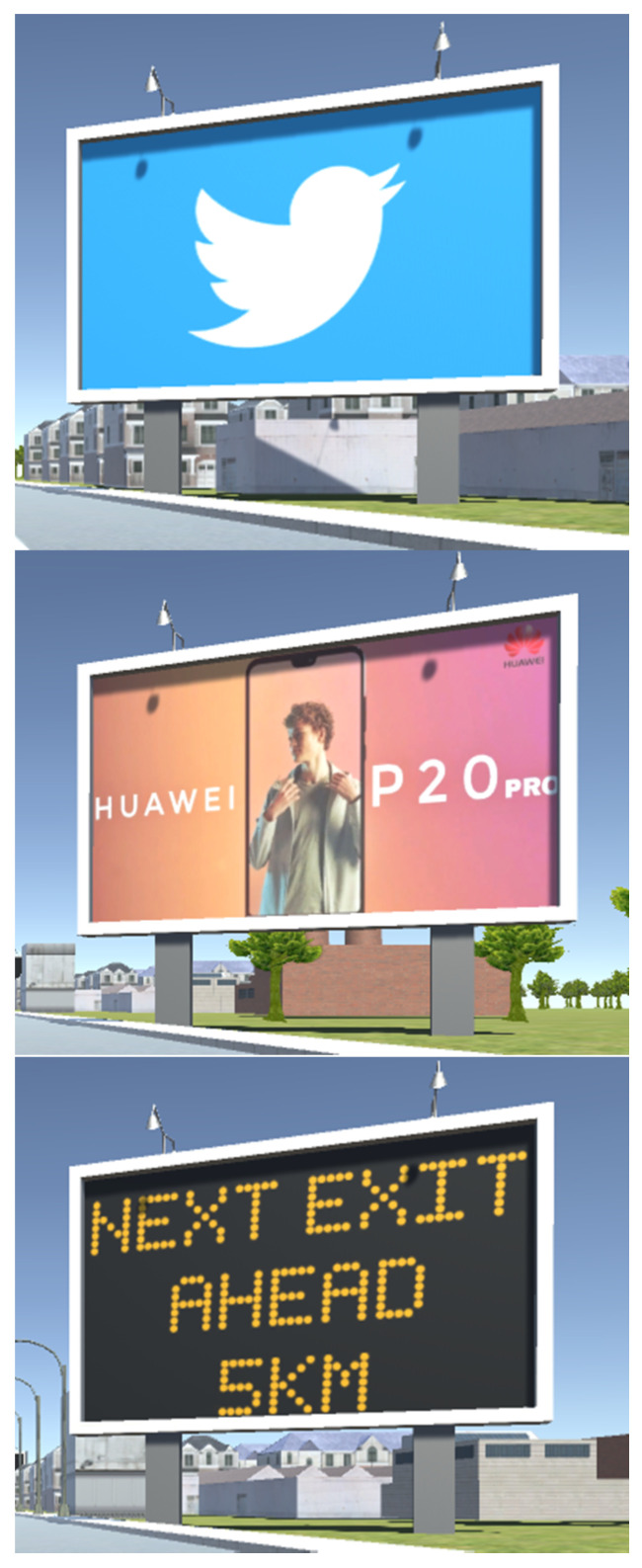
Examples of digital billboard.

The content of digital billboards was randomly chosen from well-known lists of brand logos, simple advertisements, or traffic messages. Figure 4 shows three examples of digital billboards that are used in this experiment. The 50 digital billboards have been divided into two categories. Half of the billboards are static billboards, which only display an unchanging image on the billboard throughout the experiment, the other half are dynamic billboards, which allow the displayed image that change once as the driver approaches. The dynamic billboards change their content at a distance of about 80 m from the billboard measured in the *x*-direction. This estimated threshold was defined as the distance between the point where the speed sign becomes readable to the driver and the billboard. This was done by running the simulator at a fixed speed and noting the point at which the sign was readable using a keypress.

### 2.2. Participants

Eleven healthy adults volunteered in this experiment. All volunteered participants were postgraduate students and university staff. The participants consist of 8 males and 3 females, all right-handed. The age range is from 22 to 55 years with a mean age of 32.3 years. All participants were free of past or present neurological or psychiatric conditions and with normal or correct to normal visual acuity. The ethics committee of the university approved the experiment protocol. Written informed consent was obtained prior to the experiment. After completing the measurement for all participants, 10 participants produced valid datasets for analysis.

### 2.3. Experimental Procedure

An EEG conductive gel allergic reaction test was required in advance of 24 h before the experiment, to check for any skin allergies. Prior to the experiment, all participants were asked to re-read the consent form. They were invited to ask any questions they wish and completed a short questionnaire that captures their age and level of driving experience. They were advised to take part in a practise drive to familiarise themselves with controls for the driving simulator. The experiment took place in a light dimmed, sound-attenuated, radio frequency shield room. The participants sat on a comfortable chair in front of the centre computer monitor about 100 cm away. There was a 70 cm distance between the driver’s eye and the eye tracker. A correct size EEG cap was fitted on the participant’s head with conductive gel applied. Within the ten participants, two of them were primed on the purpose of the experiment (participant number 1 and 3). All other participants were non-primed on the purpose of the experiment until the driving task was completed. Instead, they were told that the aim of the experiment was to investigate driving behaviour only. Participants were asked to obey the Irish driving regulations while completing the driving task. At the beginning of the experiment, a nine-point (a centre, four corners, and the mid-points of the four sides) eye gaze calibration step was carried out.

Each participant completed one driving run in a single recording session. The duration of one run was approximately 25 min. Four data streams were recorded for each participant during the experiment, including EEG data, eye tracking data, and camera position data in the driving simulator controls. The simulator recorded steering wheel positions and accelerator pedal positions in addition to the camera view used for rendering.

### 2.4. Data Recording

The EEG data were recorded using a g.Tec, g.USBamp device (Guger Technologies, Graz, Austria). The device uses a 16-active-electrode mounted cap and records at a sampling rate of 512 Hz. The electrode locations followed the 10–20 system montage and additional intermediate sites (AF3, F7, T7, CP5, P7, P1, PO1, Cz, Pz, PO2, P2, P8, CP6, T8, F8, AF4). The ground electrode is at AFz. The reference is at the left earlobe. In the experiment, the electrode impedance was measured and maintained under 5 kilohms using the conductive gel. A test procedure will be run to check the contact performance of the electrodes to avoid collecting contaminated data. The participants were shown the signals being recorded and asked to keep still as possible as they could to minimise the movement artefact.

Eye movement data were recorded with a portable Tobii X-120 eye tracking system. All participants had to calibrate the eye tracker using the Tobii studio software prior to the experiment start. The difference between calibration and validation measurement was kept below 1.5 degrees to ensure better eye tracking data accuracy. During the experiment, the calibration was based on the three monitors, but all eye movement tracking was associated with the centre screen. The eye tracker was used in binocular mode with data recorded with a sampling rate of 120 Hz for each eye.

The driving simulator data was recorded directly from the Unity game engine. The accelerator data was recorded from using car object controller. The billboard related event markers were also generated by the Unity game engine. The event marker was created every time the distance of the *x*-axis position of the car to the billboard falls below the threshold distance.

The billboard appearance and changing events in addition to sensor data were recorded and synchronised using a lab streaming layer (LSL) based acquisition system [47]. The LSL is a system for the unified collection of measurement time series in research experiments that handles both the networking, time-synchronization, real-time access as well as optionally the centralised collection, viewing, and disk recording of the data (see https://github.com/sccn/labstreaminglayer, accessed on 19 January 2021). The connection to the EEG system used the g.Tec application that provided by LSL. The connection to the eye tracker used a customised Python script with LSL enabled. The driving simulator data, including billboard event markers, are configured within the driving simulator application with the LSL interface program.

### 2.5. Data Analysis

**Figure 5:**
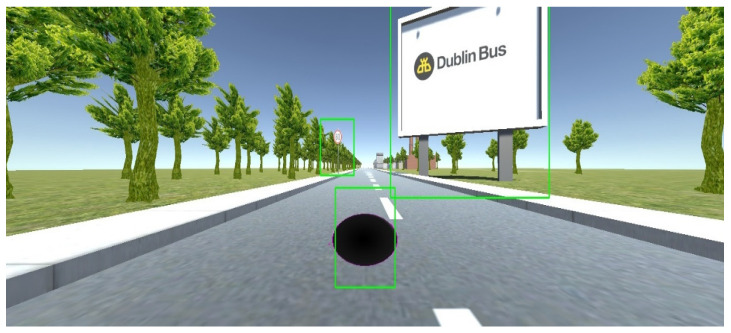
AOI bounding boxes example.

The analysis starts with dividing the entire experiment into 50 segments for each participant. Each time segment is extracted approximately 15 s before the participant drives past the billboard. Within each segment, rectangular bounding boxes are used to draw area of interest (AOI) for the speed sign, billboard and speedometer respectively to capture participant’s gaze on each of those areas, see Figure 5. The AOI for speedometer is defined approximately 3 cm larger than the actual size of the speedometer and remains the same size from beginning to end of the experiment. As participant drive the car forward, the size of speed sign and billboard increases as the vehicle moves. To make AOI track the target, the target is labelled at locations separated by 10 frames of video and intermediate values estimated by interpolation. Since the vehicle followed the same trajectory for every participant these values could be used for all participants where the recorded position of the vehicle is used to reference the driver’s view of the road. The AOI for the billboard is defined 10 cm larger than the actual size for all frames queried from playback video. This is because participants are more likely to look at the centre of the billboard than the edges.

Fixations are then extracted for all AOI labelled images using a velocity threshold of 30°/s. If the velocity associated with a gaze sample is below this threshold, then that sample is classified as belonging to a fixation. This analysis only retains fixations longer than 60 ms. Fixations with less than 0.5° apart and a time difference less than 75 ms apart are merged into a single fixation. A comparison of the total time of fixations occurring within each segment is then calculated based on four regions, speed sign, billboard, speedometer, and others (e.g., white space), followed by analysis using a *t*-test (paired two-sample of means).

Statistical analysis for the eye fixation duration uses t-test to compare the means between the dynamic segments and the static segments for each AOI.

EEG data are processed offline using EEGLAB (version 14.1.1) [48]. The data are first filtered with a finite impulse response (FIR) filter at 0.16 to 30 Hz to remove DC and high frequency noise. The filter is Hamming windowed sinc FIR filter with filter order 424. Infomax ICA is used to remove eye blink, eye movement and facial muscle movement artefacts [49]. An automatic channel rejection is used to remove bad channels. Only the electrode site P1 is removed because poor EEG signals are discovered from one participant. This still allowed grand averages to be estimated across all participants. Replacing the P1 electrode site with the average value of the nearest sites is not possible due to unbalanced electrodes around P1. EEG epochs are extracted from the continuous data between −200 ms and 800 ms with respect to the eye fixation onset time that was detected in the AOIs. To ensure that the activity is not affected by the subsequent gaze shift we retain for analysis fixations of the onset events longer than 200 ms. The eye fixation onset events were classified as follows:

- Condition (A): eye fixation on the speedometer. The following conditions must be met. Fixation on speed sign followed by a fixation on speedometer followed by speed change.
- Condition (B): eye fixation on the speedometer. The following conditions must be met. Fixation on speed sign followed by a fixation on speedometer followed by no speed change.
- Condition (C): eye fixation on the dynamic billboard. The following conditions must be met. The first valid fixation on the dynamic billboard following billboard image content change.
- Condition (D): eye fixation on the static billboard. The following conditions must be met. The last valid fixation on the static billboard before driving past the static billboard.

Each epoch is baseline corrected from −200 to 0 ms. An automatic epoch rejection is used to remove epochs, in which any voltage potential exceeded ±75 µV threshold. The number of remaining trials is used to generate a grand average analysis for each condition: condition (A), 345 trials; condition (B), 426 trials; condition (C), 89 trials; condition (D), 120 trials. Artefact-free epochs are grand averaged to reduce the random brain activity seen in the underlining ERP components following the four conditions. These include the components shown in Table 2. Differentiating the categories between billboard fixations and speedometer fixations is measured by the potential of the ERP components [50].

Statistical analysis for P1, N1, and P2 components uses two-way (fixation event, electrode site) analysis of variance (ANOVA). For the N2 component, we use an independent sample *t*-test. All analysis is conducted for the participants who are non-primed on the purpose of the experiment. No comparison of ERP data for the participants who are primed on the purpose of the experiment. This is because the grand average ERP for primed participants show a P1 component only, but no additional ERP components are observed. It is likely because the information priming has been seen to decrease activation in brain regions [51].

## 3. Results

### 3.1. Driver Eye Fixation Analysis

**Figure 6:**
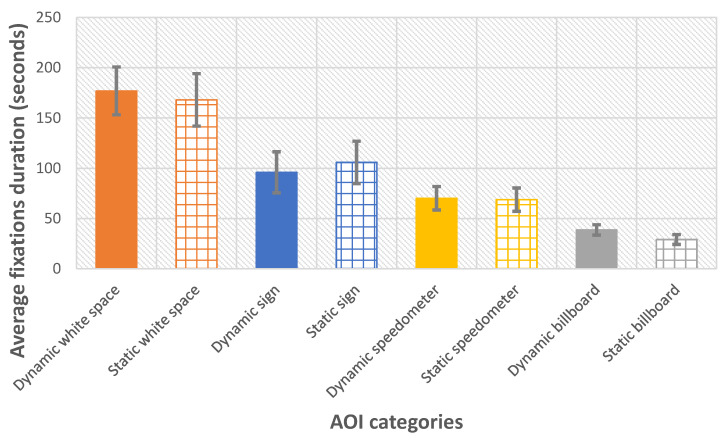
Comparison of average fixations duration in each AOI (white space AOIs, speedometer AOIs, billboard AOIs, speed sign AOIs) between dynamic segment and static segment.

The age, the driving experience, the number of dynamic billboards on which there is a fixation, and the total fixation during each AOI is shown in Table 3. The average fixation durations in each AOI between dynamic segments and static segments is shown in Figure 6.

The analysis is first conducted between the group of participants who are primed on the purpose of the experiment and the other group who are not primed. In Table 3, it shows that the participants who are primed on the purpose of the experiment have fixated on almost every dynamic billboard after they changed. This would be expected from someone with knowledge of the experiment. For this reason, all remaining participants were not informed about the dynamic change of the billboard. A difference is observed that the participants in the non-primed group have fewer fixations.

The following analysis is for non-primed participants. The number of static and dynamic billboard sequences observed by each driver was equal. In Figure 6, we calculate the averaged eye fixation duration across four AOIs between dynamic segments and static segments. Comparing the duration of the eye fixation between the dynamic billboards and the static billboards, significantly longer fixation duration is found for the dynamic billboards over those of the static billboards, by an average of 26%, t(8) = 4.41 (*p* < 0.01). This reveals that dynamic billboards take longer visual attention compares to static billboards. No significant differences are found the duration of the eye fixation between dynamic speed sign vs static speed signs, t(8) = −1.89 (*p* = 0.1), and dynamic speedometer vs. static speedometer, t(8) = 0.52 (*p* = 0.62). This shows between dynamic segments and static segments drivers were allocated an approximately equal amount of eye fixation time to speed signs and speedometers

### 3.2. Driver EFRP Analysis

**Figure 7:**
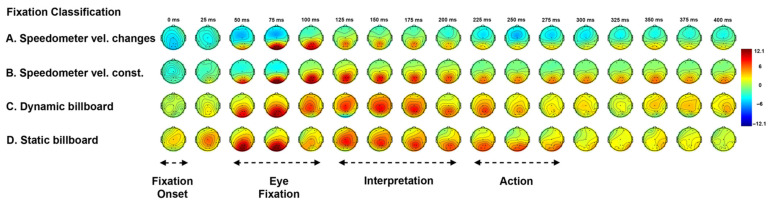
Grand average ERP in 2D topographical plot. (Condition A) Grand average ERP with stimulus event of driver fixation onset at the speedometer follow by speed changing. (Condition B) Grand average ERP with stimulus event of driver fixation onset at the speedometer without speed changing. (Condition C) Grand average ERP with stimulus event of driver first fixation on each dynamic billboard after changing the content. (Condition D) Grand average ERP with stimulus event of driver last fixation on each static billboard.

Figure 7 shows the topographical plot of the grand average ERP of the participants who are non-primed on the purpose of the experiment. As before, there are four conditions used to classify engagement. These are used to investigate the gaze related ERP signals.

#### 3.2.1. Visual P1 and N1 Components

Strong positive activity is observed at the occipital region around 75 ms after the fixation onset across all four conditions. This is referred to as the visual P1 component. During the 50 ms to 100 ms interval, two-way ANOVA analysis of peak amplitude between two factors conditions (A, B, C, D) and electrode sites (PO1, PO2) show no significant main and interaction effect (*p* > 0.1). The P1 amplitude is larger at occipital sites than other sites. For peak latency variance, there is no significant interaction effect for P1 component, F(3, 56) = 0.11 (*p* = 0.951), but different choice of conditions affect the latency, F(3, 56) = 5.93 (*p* < 0.001). Tukey’s test for post hoc analysis shows that the peak latency of condition (A) is significantly later than condition (C) (*p* < 0.05), condition (B) is significantly later than condition (C) (*p* < 0.05) and condition (D) (*p* < 0.01). The P1 latency associated with fixating on a changing billboard is earlier than on the speedometer. This signal is generated in the occipital region and so represents an early stage in processing.

**Figure 8:**
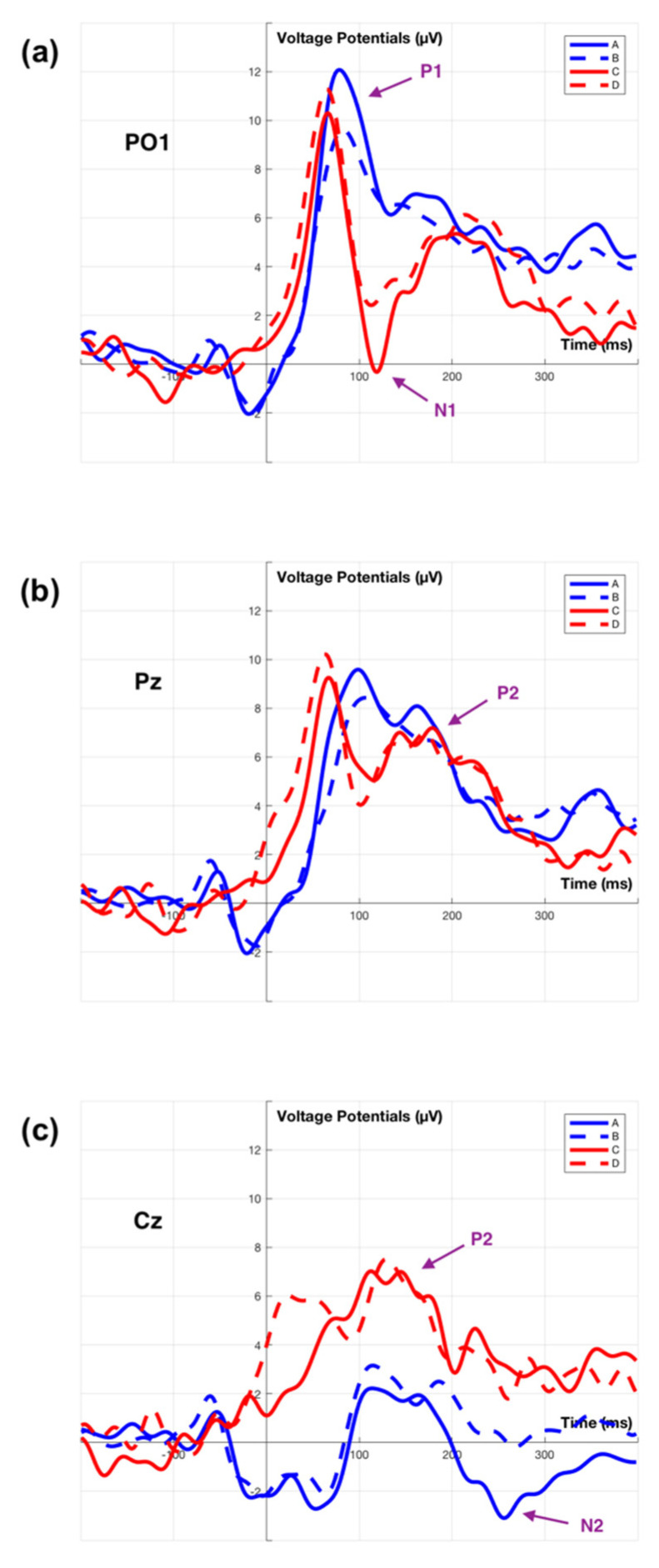
Grand average ERP of channel ERP plots. Plot (**a**). Grand average ERP of channel PO1. Plot (**b**). Grand average ERP of channel Pz. Plot (**c**). Grand average ERP of channel Cz. Signal A, B, C, D is consistent with Figure 7.

The N1 component follows the P1 component. N1 is small in the occipital sites of the topographical plot, but clearly visible in the channel ERP plot as shown in the electrode site PO1 and PO2 of Figure 8 (PO2 average ERP is not showing here). The averaged N1 peak amplitude of condition (C) is greater than condition (D), but this is not statistically significant, t(30) = −1.401 (*p* = 0.172). In the electrode site PO1, the waveforms are different between conditions using the fixation on speedometer (A,B), and conditions using the fixation on billboards (C,D). The peak amplitude during the 75 ms to 125 ms interval shows no significant interaction effect between conditions (A, B, C, D) and electrode sites (PO1, PO2), F(3, 56) = 0.04 (*p* = 0.989), but reveals a significant main effect of conditions, F(3, 56) = 7.92 (*p* < 0.001). Tukey’s test for post hoc analysis shows that condition (A) is significantly greater than condition (C) (*p* < 0.001) and condition (D) (*p* < 0.05), condition (B) is significantly greater than condition (C) (*p* < 0.01). In brief, this shows a significant N1 (amplitude) component for billboard interaction when compared to the speedometer. There is no significant main and interact effect found for N1 latencies (*p* > 0.1).

#### 3.2.2. P2 Component

In Figure 7, a posterior P2 component is observed between 150 ms to 225 ms. Comparing between condition (A,B), speedometer fixation, and condition (C,D), billboard fixation, the P2 component shows a weaker potential for condition (A,B), and appears in the parietal region and fades in the occipital region. For condition (C,D), the P2 component shows stronger potential and has a much large affect area in the central region, then fades in the parietal-occipital region. The two-way ANOVA of posterior P2 amplitude is carried out across parietal sites (electrodes Pz, P2) and central site (electrode Cz) reveals significant main effect for both electrode sites, F(2, 84) = 6.94 (*p* < 0.01), and conditions, F(3, 84) = 3.24 (*p* < 0.05). Tukey’s test for post hoc analysis shows only condition (B) is significantly different to condition (C) (*p* < 0.05), and the central site is significantly different to parietal sites (*p* < 0.01). No interaction between electrode site and conditions is observed. Overall, P2 latency shows no significant interaction effect, but main effect of conditions is significant, F(3, 84) = 3.21 (*p* < 0.05). Tukey’s test for post hoc analysis shows condition (A) is different from condition (C) (*p* < 0.05). In summary, the P2 is present in both the speedometer and billboard fixations located in the parietal region. There is an increased potential for the P2 produced by the fixation on the dynamic billboard transition when compared to a speed sign fixation.

#### 3.2.3. N2 Component

For condition (A) in Figure 7, a negative deflection is observed at 250 ms. This is referred to as the N2 component. This negative only appear within the condition (A) is related to the driver’s speed adjustment after fixation on the speedometer. In other conditions, there is no such negative deflection. Welch’s ANOVA analysis conducts at the 225 ms to 275 ms interval reveals a significant potential difference of conditions. Welch’s correct F ratio is F(3, 13.72) = 4.59 (*p* < 0.05). The N2 peak latency with standard error for condition (A) is 247.07 ± 4.92 ms (SD = 13.93).

## 4. Discussion

From the literature, we know there are a substantial number of road accidents that are related to distractions external to the vehicle [52]. In this research, we investigate driver visual and cognitive responses, which are influenced by digital billboards in a driving simulator. The measurement of fixation time depends on the number of fixations and the duration of each fixation summed over all trials.

The total fixation duration for billboards is the shortest duration. This demonstrates that the drivers spend less time attending to the distraction offered by the billboard than other tasks. Comparing dynamic billboard fixation duration and static billboard fixation duration, the results show that there is a significantly longer fixation duration on dynamic billboards than static billboards. This finding is supported by research studies examining the impact of billboards on driver distraction [19,21,25].

Controlling the speed of the car requires the driver to look at both the speed sign and the speedometer to arrive at the correct speed. The fixation time associated with reading the speed signs is longer than for the speedometer. This might be due to the fixed location of the speedometer simplifying the task of reading it. Across both static and dynamic segments, the percentage of time spent fixating on the speedometer was 18.4% and on the speed sign was 26.8% in regions where billboards and signs were present. The total time spent on fixations required to control the speed of the car was 45.2%. The total time fixating on billboards was 9.0%. The trial periods used to measure the billboard engagement occupied about 40% of the total drive time. The work has demonstrated that the billboards engage the driver’s attention, during the important times when the speed needs to be adjusted. It would require further work to assess the impact on the overall driving performance. Some white space fixation might be used for estimating the speed of the car by tracking features in the scene, this was not included in the value.

A preliminary observation that can be obtained from the data in Table 3 is that there is a correlation between the age group and the level of engagement with dynamic billboards. The data suggests that as age increases the time spent looking at the billboard might increase. This observation was made by comparing two groups above and below the age of 26 years. This finding is consistent with a recent study, which investigates the prevalence of secondary-task engagement and its impact on driver distraction and crashes. The work reveals that older drivers whose ages range from 30 to 64 are distracted by the events external to the vehicle at 1.04% of the time, more than younger drivers whose ages range from 21 to 29 at 0.82% of the time [53]. A larger cohort and extended study would be required to confirm this observation.

This present study provides an investigation of the EEG activity between normal driving and distracted driving associated visual attention measurements (e.g., speedometer and digital billboard) in a simulated driving environment using EFRP. In the research area of driver distraction, there are only a few studies that assess driver cognitive response using ERP even fewer using EFRP. Most research studies that are using the EFRP technique, limit the complexity of visual stimuli, such as using a single letter [54] or simple images [55]. More importantly, those stimuli are often placed in the centre of the display to minimise gaze shifts [56]. More advanced EFRP studies allow users to free search in large single static images [57,58,59]. These studies do not reveal the user’s eye fixation brain potential in a simulated environment. Renold et al. [42] published a study measuring drivers EFRP. The study required the driver to identify target stimuli and so produced ERP signals using a primed driver.

In this research, we extend the approach of EFRP analysis applied in most research to date, to study driver visual and cognitive responses to safe driving in a dynamic environment. Drivers are instructed to freely explore the scene just as normal driving. In contrast to [42], we do not give any specific task to the driver while performing the driving task. There was no priming of the driver about the stimuli being used to create an ERP signal, so that the driver’s psychophysiological status in real driving scenarios is likely to be restored in the experiment.

**Figure 9:**
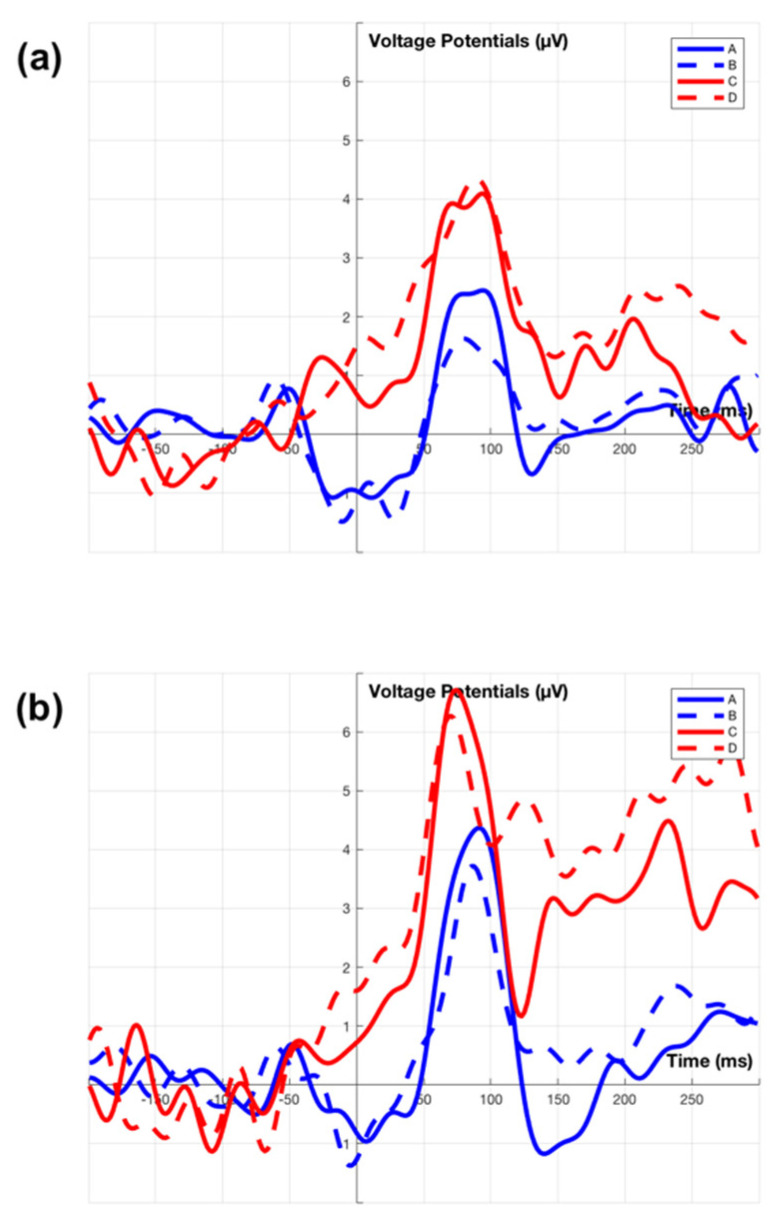
Grand average ERP of channel ERP plots. Plot (**a**). Grand average ERP of channel P7. Plot (**b**). Grand average ERP of channel P8. Signal A, B, C, D is consistent with Figure 7.

The experimental results demonstrate similarities and differences of evoked EFRPs, when the driver fixates on the speedometer (with speed adjustment and without speed adjustment) and billboards (dynamic billboards and static billboards). A common signal between these two categories of fixations is a first positive activity (P1 component) over the occipital region at about 50 ms to 100 ms after fixation onset. The occipital P1 (and subsequent N1) component is sensitive to visual stimulus factors and index early sensory processing (e.g., perceptual analysis) [60,61]. This early component is reliably modulated by voluntary spatial attention [60,62]. Unlike the lateral occipital region elicited P1 component from either stimulus discrimination task [63] or selective attention task [64], the present finding of the P1 component only appears at the central occipital region, represent a registration of visual stimuli in the participant’s brain. In this experiment, the P1 appears in reconstruction near the midline this is in part due to the electrode placement. In practice, others report the maximum P1 signal lateral to the midline. Apart from these centrally located electrodes, there are two electrodes located at P7 and P8. The same ERP analysis is also carried out for P7 and P8 across the two categories as shown in Figure 9. We observe the right hemifield (P8) elicits a larger P1 component than the left hemifield (P7). The result is contradictory to other findings that show a contralateral early positivity response in the brain following visual stimuli [65,66,67]. Although the present study uses driver fixation data over the right-side billboards and bottom side speedometer, the complexity of visual stimuli exceeds those previous experiments.

The first divergence between the two categories of fixation is the negative activity (N1 component) over the occipital region at about 75 ms to 125 ms after fixation onset. The N1 component reveals an amplitude difference between the fixations on the speedometer and billboards as shown in Figure 8. The N1 components are influenced by spatial attention, where larger amplitude (more negative) is associated with stimuli that appear at an attended location than an unattended location [62]. There is a possibility that the driver will learn that some billboards are likely to change as they approached. Furthermore, the amplitude of N1 can also be affected by the level of attention [68]. The larger N1 amplitude that is obtained by fixating on dynamic billboards may be due to the driver’s expectation of the dynamic billboards change. In contrast to the speedometer, which existed at the bottom centre of the screen throughout the entire experiment, the eye fixation on the speedometer should not belong to either attended or unattended stimuli.

The second divergence between the two categories of fixation is the positive activity (P2 component) over the central–parietal region at about 150 ms to 225 ms after fixation onset. The characteristics of the P2 component are associated with the secondary processing of visual input and reflect the general neural processes of comparing visual input with internal expectation in memory [69,70,71,72]. The larger P2 amplitude is observed for fixation on billboards rather than speedometers. This suggests that the size of the stimuli affect the visual information processing signal [73]. Apart from the P2 component, a wider spectrum for the posterior P1 component appears in the topographical plot (Figure 7) and the larger N1 component appears in the average ERP plot (Figure 8) are also likely to be influenced by the size of the stimulus [74,75].

The maximum potential moves from the occipital lobe towards the central parietal lobe over a period of 50 ms starting 50 ms after stimulus onset, see Figure 8. This reflects the passage of the image through different processing functions within the brain as an image is interpreted.

In addition to the ERP components mentioned above, there are several additional findings that may have the potential to act as a criterion for analysing driver distraction. Firstly, a negative activity (N2 component) over the central region at about 225 ms to 275 ms after the fixation on the speedometer, e.g., condition (A). Although this condition is deliberately selected from the fixation on the speedometer so as to allow a comparison with the other conditions, e.g., condition (B).

**Figure 10:**
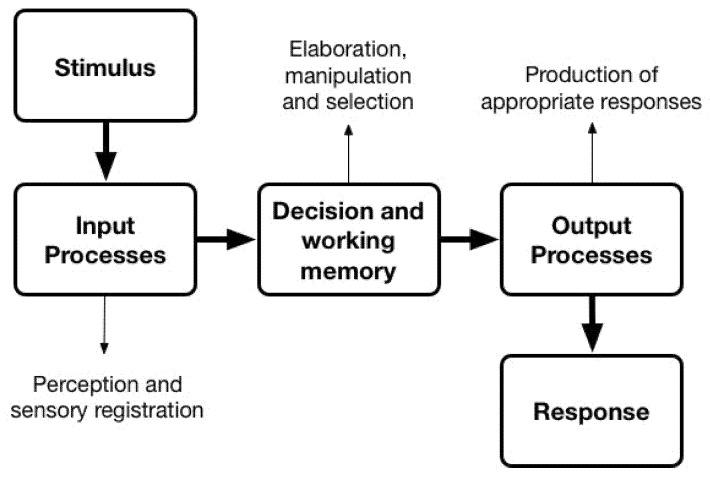
A basic human information processing model (figure is adapted from [77]).

Furthermore, we can show the series of ERP components agrees with several proposed human information processing models, for example, Figure 10. The input processes stage refers to the acquisition and registration of multiple sources of information [76]. The visual P1, N1 components represent the registration of stimulus input. The following stage, decision, and working memory, involves conscious perception and manipulation of processed and retrieved information in working memory, in addition, this stage also includes cognitive operations, but these operations occur prior to the point of decision, then decisions are made based on such cognitive processing [76]. The P2 component represents the early cognitive process to the input stimulus. The output processes stage involves the implementation of a response or action consistent with the chosen decision [76]. The N2 component reveals the production of appropriate responses. The analysis has demonstrated that the combination of ERP and eye tracking, when used in a driving simulator, can be utilised to follow the cognitive response related to driving stimuli as they are processed by the brain.

One unexpected finding in the present study appears a review of the conversation after the completion of the experiment, that six participants (80%) report they cannot ignore a specific billboard within the driving simulator experiment. The goal of advertising is to get people’s attention which this specific billboard achieved. There is likely to be a balance between the level of engagement that a specific billboard demands and the related impact on driver attention. When an individual encounters novel things or in new external conditions, entropic imagery, they will have the psychological tendency to pay increased attention to it. Future research may focus on using scientific methodologies to find an optimal balance between driver distraction and digital billboards for the purpose of safe driving.

## 5. Conclusions

This paper has described the development of a driving simulator platform that includes a synchronous recording of eye tracking, EEG, and driver motor response. The apparatus was used to record data from participants viewing static and dynamic billboards. The cognitive responses generated by the stimuli were extracted from the data. The analysis shows increased cognitive activity related to dynamic billboards. The level of engagement with the billboard is likely to be a precursor to driver distraction. This paper has shown that it is possible to analyse this step in the distraction process and demonstrates the functionality of the proposed measurement system as a valid tool in assessing driver cognitive responses to billboards.

## Figures and Tables

**Table 1:** Unity driving simulator parameters quantifying speed signs and digital billboards.

Speed Limit	Number of Speed Signs	Distance Between Current Sign to Next Sign
40	18	300
60	13	400
80	19	500

**Table 2:** Selection of cortical regions that used to measure the ERP components.

Cortical Regions	Available Electrodes	Measured ERP Component	Component Peak Latency Range
**Parietal–Occipital**	PO1, PO2	P1	50–100 ms
**Parietal–Occipital**	PO1, PO2	N1	75–125 ms
**Central–Parietal**	Pz, P2, Cz	P2	150–225 ms
**Central**	Cz	N2	225–275 ms

**Table 3:** Eye gaze fixation duration (seconds) of each individual participant, from approximately 15 s before the car driving pass by the billboards. Fixations are measured between static billboards and dynamic billboards across four AOIs, white space, speedometer, billboard, and speed sign.

Participant Number	Age Group *	Driving Experience *	Number of Distractions **	Dynamic White Space	Static White Space	Dynamic Speedometer	Static Speedometer	Dynamic Billboard	Static Billboard	Dynamic Speed Sign	Static Speed Sign
1	51–55	30	24	181.62	167.64	45.16	42.33	56.21	31.09	77.51	105.27
2	41–45	26	21	117.10	113.65	86.63	79.82	57.74	43.40	79.39	104.28
3	26–30	<1	25	97.97	101.73	143.60	153.17	67.78	70.30	100.89	82.46
4	26–30	7	19	285.48	291.37	29.02	42.86	25.71	10.08	2.99	6.55
5	26–30	3	11	172.27	152.77	63.90	63.96	29.00	19.43	139.56	149.64
6	26–30	9	15	147.88	135.79	85.18	78.48	47.76	39.50	101.48	129.06
7	21–25	6	9	201.25	182.38	108.35	105.27	32.28	30.33	48.83	42.94
8	21–25	5	8	99.78	87.16	104.08	108.30	38.15	20.58	125.07	151.52
9	21–25	<1	4	259.48	263.78	17.80	8.37	19.91	19.13	77.90	79.18
10	26–30	7	2	131.48	116.69	65.54	62.35	57.73	49.51	191.84	182.92
Mean (all)	-	-	13.80	169.43	161.30	74.93	74.49	43.23	33.33	94.55	103.38
SD (all)	-	-	8.23	64.42	68.26	38.57	40.81	16.37	17.89	51.40	53.23
SE (all)	-	-	2.60	20.37	21.59	12.20	12.91	5.18	5.66	16.26	16.68
Mean (u)	-	-	11.13	176.84	167.95	70.06	68.68	38.53	28.99	95.88	105.76
SD (u)	-	-	6.79	67.24	73.67	32.95	32.72	14.46	13.93	57.86	59.78
SE (u)	-	-	2.40	23.77	26.05	11.65	11.57	5.11	4.92	20.46	21.13

- Mean (all), SD (all), SE (all) = average fixations, standard deviation and standard error across all participants. - Mean (u), SD (u) = average fixations, standard deviation and standard error across participants who are non-primed on the purpose of the experiment. (Participants number 2, 4, 5, 6, 7, 8, 9, 10). * The units of age group and driving experience are years. ** The number of dynamic billboards on which there is a fixation

## Data Availability

All data is anonymised and stored at Maynooth University following the rules set out by the Maynooth University Data Protection Office.
